# Preliminary study on CT contrast-enhanced radiomics for predicting central cervical lymph node status in patients with thyroid nodules

**DOI:** 10.3389/fonc.2023.1060674

**Published:** 2023-02-03

**Authors:** Dan Kong, Wenli Shan, Yan Zhu, Qingqing Xu, Shaofeng Duan, Lili Guo

**Affiliations:** ^1^ Department of Imaging, The Affiliated Huaian No. 1 People’s Hospital of Nanjing Medical University, Huaian, Jiangsu, China; ^2^ Institute of precision medicine, GE Healthcare, Shanghai, China

**Keywords:** thyroid, cancer, lymph node, radiomics, x-ray computed tomography, contrast enhancement

## Abstract

**Objective:**

To explore the feasibility of using a contrast-enhanced CT image-based radiomics model to predict central cervical lymph node status in patients with thyroid nodules.

**Methods:**

Pretreatment clinical and CT imaging data from 271 patients with surgically diagnosed and treated thyroid nodules were retrospectively analyzed. According to the pathological features of the thyroid nodules and central lymph nodes, the patients were divided into three groups: group 1: papillary thyroid carcinoma (PTC) metastatic lymph node group; group 2: PTC nonmetastatic lymph node group; and group 3: benign thyroid nodule reactive lymph node group. Radiomics models were constructed to compare the three groups by pairwise classification (model 1: group 1 vs group 3; model 2: group 1 vs group 2; model 3: group 2 vs group 3; and model 4: group 1 vs groups (2 + 3)). The feature parameters with good generalizability and clinical risk factors were screened. A nomogram was constructed by combining the radiomics features and clinical risk factors. Receiver operating characteristic (ROC) curve, calibration curve and decision curve analysis (DCA) were performed to assess the diagnostic and clinical value of the nomogram.

**Results:**

For radiomics models 1, 2, and 3, the areas under the curve (AUCs) in the training group were 0.97, 0.96, and 0.93, respectively. The following independent clinical risk factors were identified: model 1, arterial phase CT values; model 2, sex and arterial phase CT values; model 3: none. The AUCs for the nomograms of models 1 and 2 in the training group were 0.98 and 0.97, respectively, and those in the test group were 0.95 and 0.87, respectively. The AUCs of the model 4 nomogram in the training and test groups were 0.96 and 0.94, respectively. Calibration curve analysis and DCA revealed the high clinical value of the nomograms of models 1, 2 and 4.

**Conclusion:**

The nomograms based on contrast-enhanced CT images had good predictive efficacy in classifying benign and malignant central cervical lymph nodes of thyroid nodule patients.

## 1 Introduction

Thyroid nodules are a common disease that appear in the neck, the most common of which are papillary carcinoma (PTC) and adenoma, which are often associated with enlarged lymph nodes in the neck. Patients with PTC have a relatively good prognosis, with a mortality rate of less than 10% (1, 2). However, approximately 40-70% of patients with PTC develop cervical lymph node metastases, which are associated with recurrence and poor prognosis (3). Lymph node metastasis occurs mainly in the central region, which contains the sentinel lymph nodes of thyroid cancer, while the lymph nodes of adenoma are mostly reactive hyperplasias. Some patients present with enlarged lymph nodes in the neck, and therefore, the nature of the lymph nodes often needs to be determined in the absence of a clear etiology. Currently, the analysis of lymph nodes mainly relies on morphology, but enlarged lymph nodes may be attributable to inflammatory reactive hyperplasia, while normal-sized lymph nodes may also have tumor infiltration; therefore, the accuracy in assessing benign and malignant lymph nodes based solely on morphological features (e.g., size) is not high (4), with an overall misclassification rate of approximately 15%-20% (5). Radiomics can help identify features that are difficult to observe with the naked eye in images and can be used to quantitatively assess the heterogeneity of lesions (6). Recent studies have found that radiomics can be used to describe tumor phenotypes, distinguish benign and malignant tumors, and predict lymph node metastasis and outcomes (7). Radiomics models have been shown to predict lymph node metastasis in PTC (8, 9), and CT-based radiomics models are also valuable in the differentiation of benign and malignant lymph nodes in the head and neck (10). Few studies have examined the differentiation of metastatic and nonmetastatic lymph nodes among patients with PTC and the classification of reactive hyperplastic lymph nodes among patients with benign thyroid lesions. The purpose of this study was to determine the efficacy of using a CT-based radiomics model to classify lymph nodes in the central neck regions of patients with thyroid nodules.

## 2 Materials and methods

### 2.1 General information

This retrospective study was approved by a hospital ethics committee, and informed consent was waived. We retrospectively collected data from patients with surgically and pathologically confirmed thyroid nodules from May 2020 to December 2021. Clinical data and contrast-enhanced CT images of lymph nodes in the central cervical region were collected. The inclusion criteria were as follows: 1. A single thyroid lesion, diagnosed as PTC or thyroid adenoma by postoperative pathology; 2. intraoperative lymph node dissection in the central neck region revealing a PTC pathology (for patients with PTC) or no lymph node pathology (for patients with thyroid adenoma); no history of malignant tumors or blood disorders; and normal tumor-related indicators on preoperative routine examination; 3. lymph node diameter in the central region of the neck ≥5 mm (11); 4. contrast-enhanced CT examination performed within two weeks before surgery and treatment consistent with the diagnosis and postprocessing; and 5. no radiotherapy or chemotherapy before surgery. To avoid experimental deviation, lymph nodes larger than 20 mm were excluded from this study.

For the final lymph node pathological findings, the all-or-none principle was used (12). When all lymph nodes within the central zone of PTC patients had metastatic pathological findings, the lymph nodes seen on images within the zone were labeled metastatic lymph nodes; when all lymph nodes within the zone had nonmetastatic pathological findings, the lymph nodes seen on images within the zone were labeled nonmetastatic lymph nodes; and when the lymph node pathological results were both metastatic and nonmetastatic, they were not included. One large and clearly displayed lymph node in the central region was selected as the target lesion for each patient.

**Figure 1:**
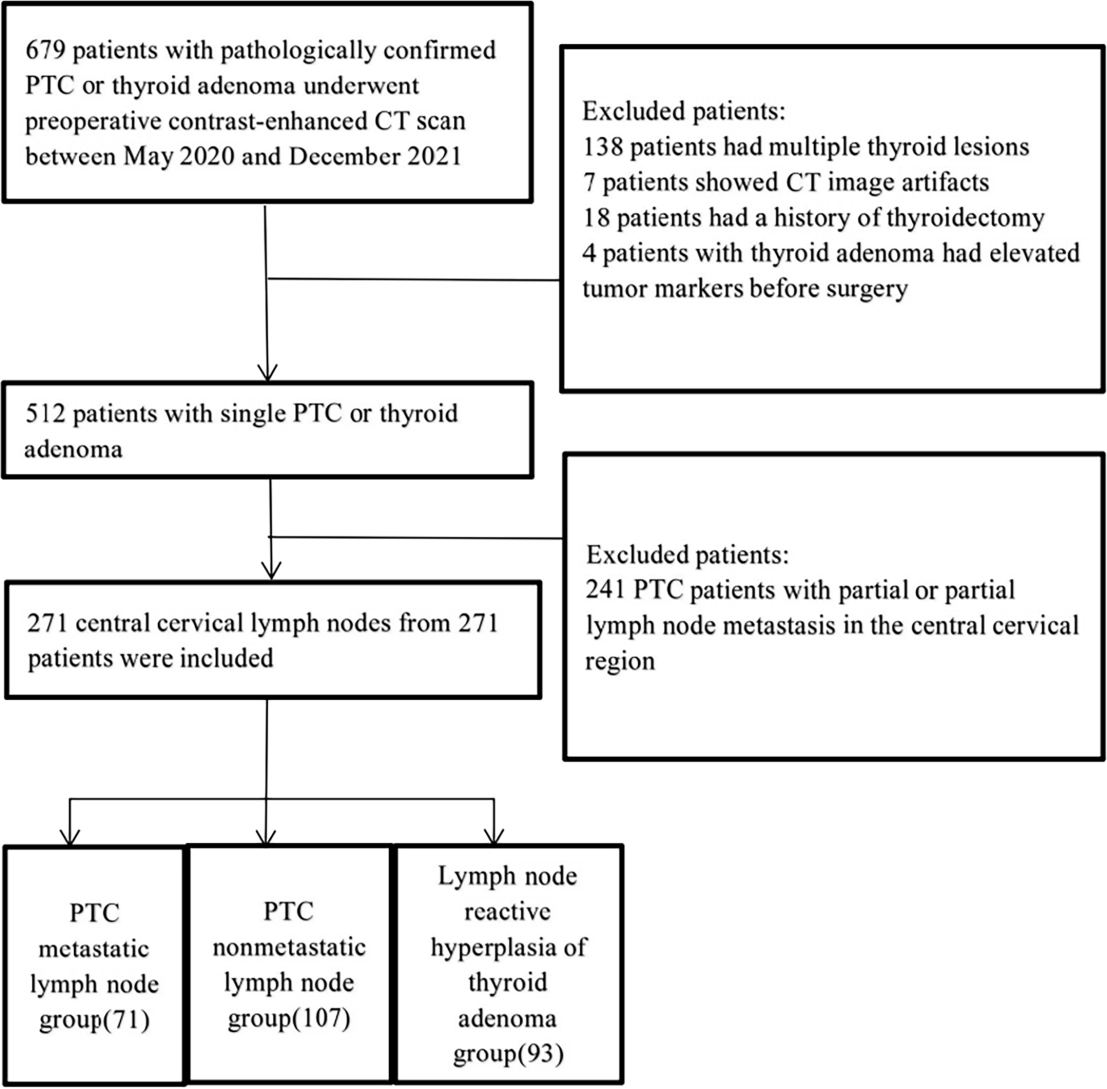
Patient enrollment process.

**Figure 2:**
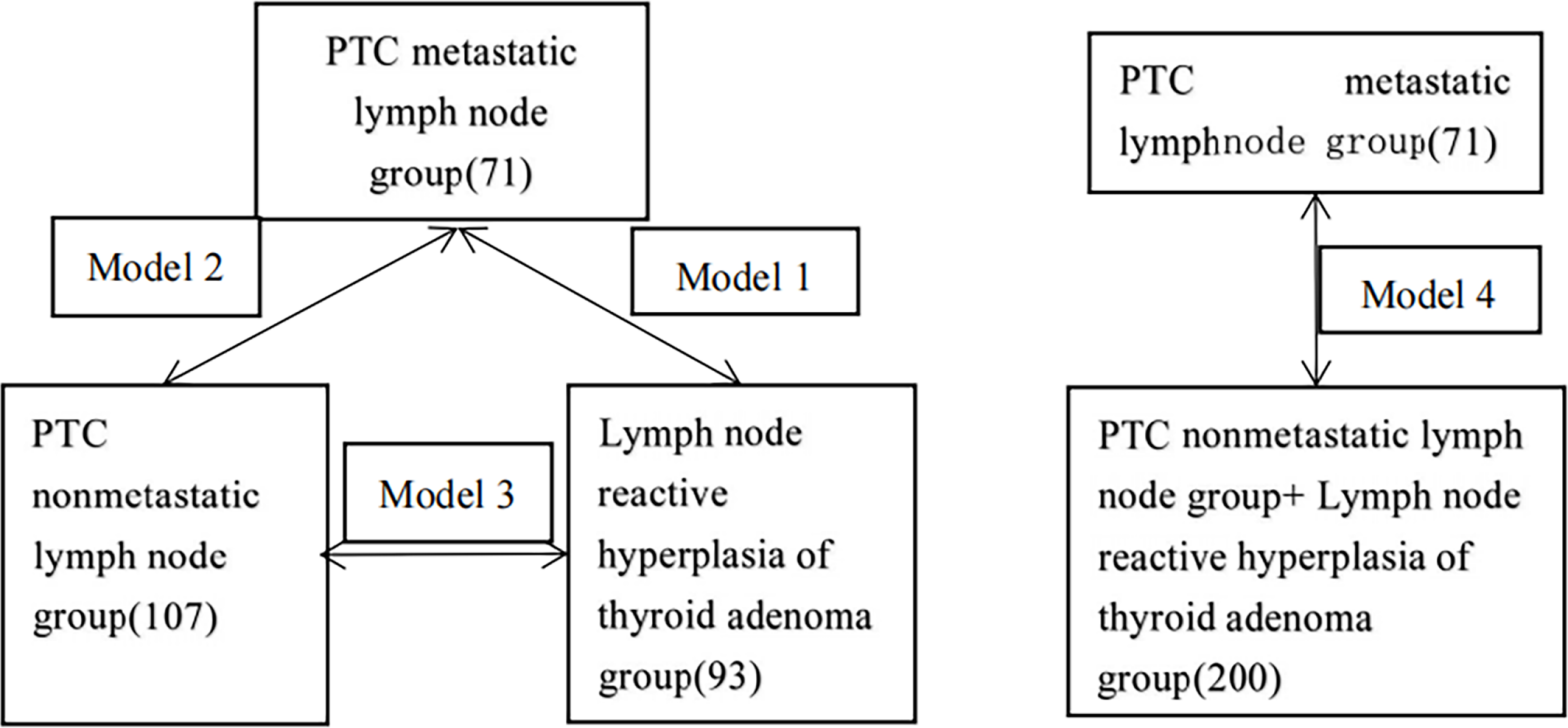
Schematic diagram of model construction.

A total of 271 patients were included in this study, including 41 males and 230 females, with ages ranging from 22-78 years (average 46.5 ± 17.4 years). The flowchart of inclusion and exclusion is shown in **Figure 1**. A total of 178 patients had PTC (71 patients in the lymph node metastasis group and 107 patients in the nonmetastasis group). Ninety-three patients were pathologically diagnosed with thyroid adenoma, and their lymph nodes were classified into the benign reactive lymph node group. Based on the pathologic findings of thyroid nodules and central lymph nodes, we divided the patients into three groups: the PTC metastatic lymph node (MLN) group; the PTC nonmetastatic lymph node (non-MLN) group; and the hyperplastic lymph node (HLN)/thyroid adenoma group. Four classification models were constructed, as shown in **Figure 2**.

First, three models were constructed:

Model 1: MLN 71 patients, HLN 93 patients;
Model 2: MLN 71 patients, non-MLN 107 patients;
Model 3: non-MLN 107 patients, HLN 93 patients.

Second, model 4 was constructed, including 71 patients in the MLN group and 200 patients in the HLN and non-MLN groups.

### 2.2 CT examination method

A Siemens definition 64-slice CT scanner from Germany was used to perform routine noncontrast scans and 2-phase enhanced scans. The tube voltages were 120 kV, and CARE Dose 4D was used. The slice thickness was 2 mm, and the pitch was 0.8. A volume of 60-70 ml of the contrast agent ioversol (containing 320 mg/ml iodine) was injected into the median elbow vein at an injection rate of 3.0 ml/s, followed by injection of 15 ml of normal saline. The aortic arch was monitored by the contrast agent bolus tracking method, with a trigger threshold of 100 HU, an arterial phase delay time of 10 s, and a venous phase delay of 25 s. Before the scan, the patients were instructed to breathe and then hold their breath with the arms placed at the sides of the body. During the scan, the patients were asked to maintain a supine position with the neck leaning backward and maximally lowered shoulders, and swallowing was prohibited. The scanning range extended from the mandible to the base of the neck. If the thyroid extended behind the sternum, the scanning range was expanded to cover the entire thyroid.

### 2.3 CT image analysis

The clinical and imaging data of all patients were analyzed by two experienced radiologists to determine lymph node status, including age, sex, the short and long diameters of the lymph nodes in the transverse axis on the slice showing the largest area, the shape of the lymph nodes, the CT value during the arterial and venous phases, and the difference in the CT value between the venous phase and arterial phase. The CT value is a measurement unit that can reflect the density of the lymph nodes. Two experienced radiologists measured the corresponding CT values three times and took the average value. During the measurement, the radiologists ensured that the solid area was as wide as possible, and cystic necrosis and calcification was avoided as much as possible. Any obvious differences between the radiologists’ evaluations were resolved by consensus.

### 2.4 Region of interest segmentation and feature extraction

#### 2.4.1 ROI segmentation

Two experienced radiologists (Doctor A and B) used ITK-SNAP (www.itksnap.org) software to delineate the lymph node edges layer by layer on the arterial- and venous-phase CT images to synthesize a 3-dimensional (3D) ROI. Doctor A performed two delineations, 1-2 weeks apart; Doctor B performed one.

#### 2.4.2 Radiomics feature screening and establishment of the radiomics model

The arterial- and venous-phase images were standardized using A.K software (version: 3.2.0.r, Artificial Intelligence Ki, GE Healthcare), and then, the radiomics features were extracted from the 3D ROIs using PyRadiomics. We used R software (http://www.Rproject.org, version 3.4.4) to analyze the data. After lymph node segmentation, the software automatically obtained 14 shape features, 18 first-order features, and 68 texture features. We first performed consistency tests within and between the observer datasets; that is, we calculated the intraclass correlation coefficient (ICC) between the features extracted from the two ROIs constructed by Radiologist A as well as the ICC between the features extracted from the first ROI constructed by Radiologist A and that constructed by Radiologist B. Features with an ICC>0.75 in both calculations were retained, and the features extracted from the first ROI constructed by Radiologist A were used for subsequent analysis.

**Figure 3:**
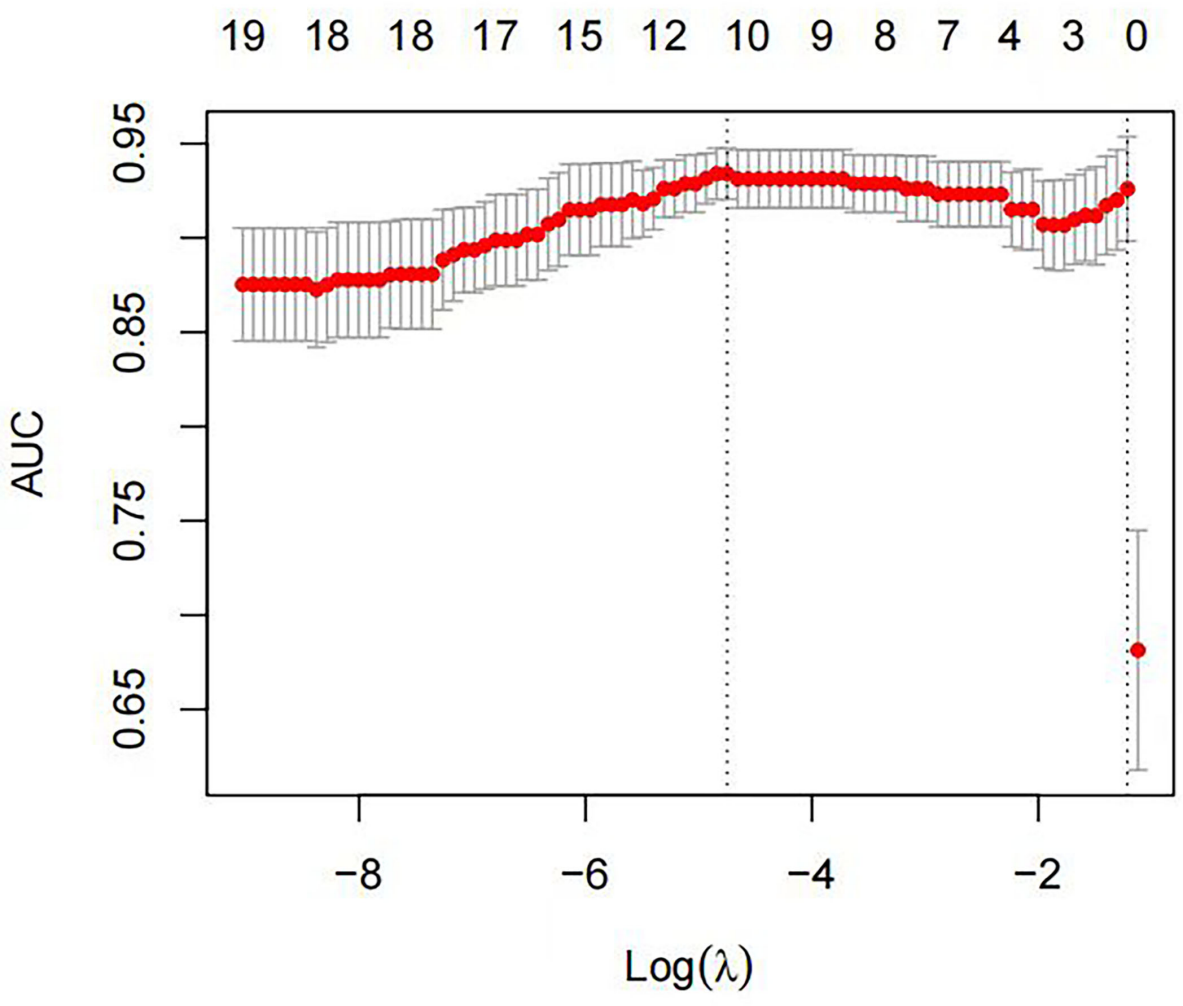
The optimal tuning parameter (λ) was selected using ten-fold cross-validation with the LASSO regression model. The horizontal axis represents the log value of the best λ, and the vertical axis represents the corresponding binomial deviation value. The red dots represent the average deviation of a given λ value, the corresponding vertical line represents the upper and lower limits, and the dotted lines represent the selected optimal log(λ) values.

**Figure 4:**
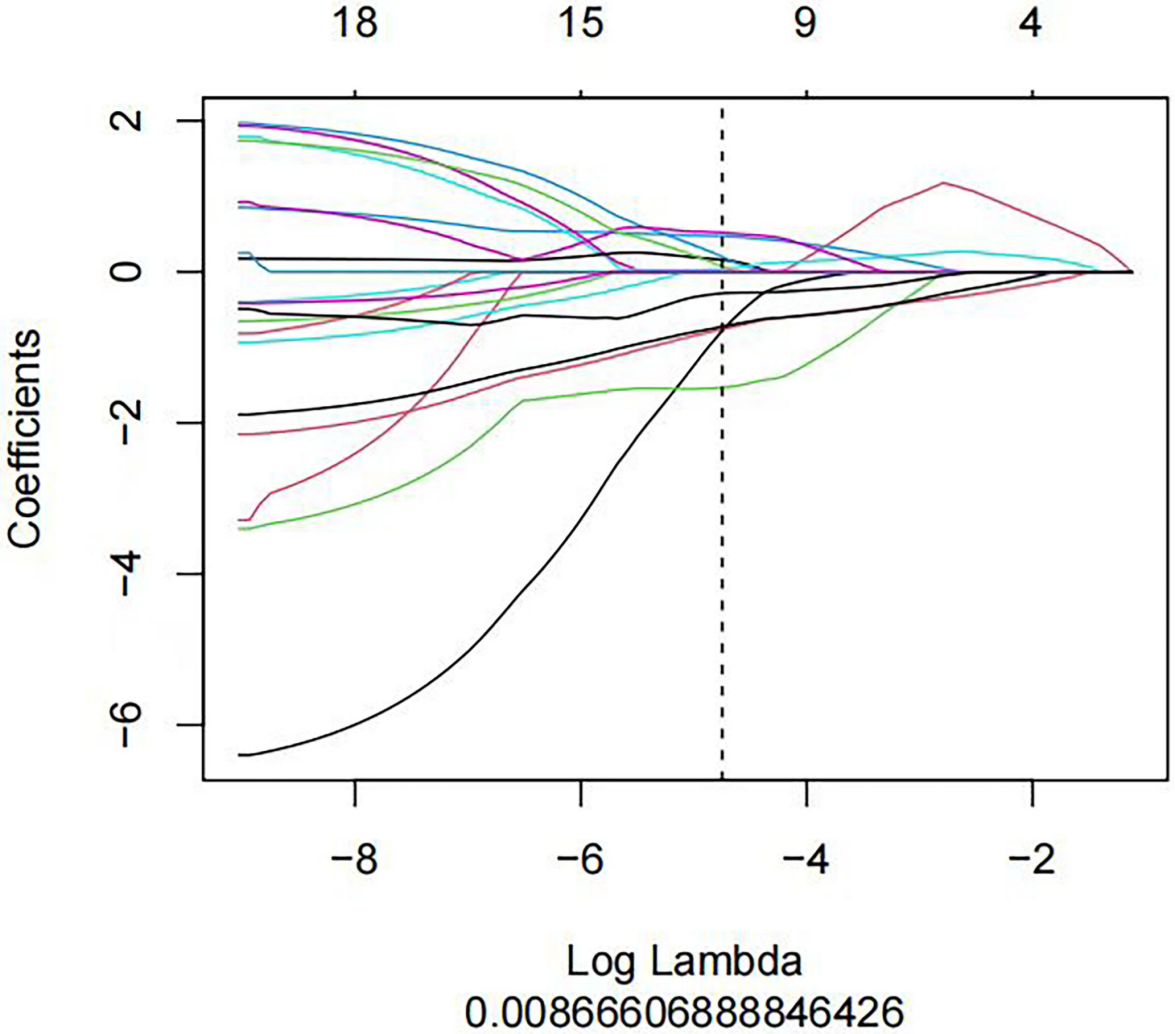
A LASSO regression model was used to screen out the profile of radiomics feature coefficients in each model. The horizontal axis represents log(λ), and the vertical axis represents the selected characteristic coefficients. Each line represents the trend for each feature.

We used the stratified random sampling method to divide patients into training and test groups at a 7:3 ratio. The training group data were used for feature screening and model construction. First, maximum relevance and minimum redundancy (mRMR) were used to remove redundant and irrelevant features, ultimately retaining 30 features, which were then imported into the least absolute shrinkage and selection operator (LASSO) regression model. Ten-fold cross-validation was used to identify the hyperparameter λ of the LASSO regression model; the value corresponding to the minimum model error was selected to retain features with nonzero coefficients (**Figures 3**, **4**). Regression and dimensionality reduction were used to further select features with good generalizability, which were then used to establish a prediction model. Each lymph node was scored according to the weights of the screening features. A diagnostic model was established by machine learning using the subset of the data screened by the feature variables, and the validity and reliability of the diagnostic model were evaluated by using the area under the receiver operating characteristic (ROC) curve (AUC). The characteristics of good repeatability and stability were used to build the radiomics model. Linear fusion of the selected features was performed to calculate the radiomics score.

### 2.5 Construction and evaluation of the radiomics nomogram

In the training group, the clinical risk factors were screened by one-way ANOVA and then were further analyzed by multivariate logistic regression analysis to finally determine the independent clinical risk factors (*P*< 0.05), which were then used to construct the clinical models. A nomogram was established by combining the radiomics signature and the clinical risk factors.

The predictive efficacy of the nomogram was assessed using ROC and calibration curve analysis. The DeLong test was used to compare the AUCs between different variables. The calibration curve reflects the agreement of the predicted probability of the nomogram with the pathological diagnosis; the closer the calibration curve is to the diagonal line, the closer the predicted value of the model is to the true value, and thus the better the calibration is. Decision curve analysis (DCA) was used to evaluate the potential net clinical benefit and utility of the prediction model and to validate it in the test group.

### 2.6 Statistical analysis

All data were statistically analyzed using SPSS 24.0 and R3.4.4 software (https://www.Rproject.org). Single-factor ANOVA was used to compare patient age between the 3 groups, the LSD method was used for two-way comparisons, and the χ² test was used to compare differences in sex distributions. Otherwise, the Mann-Whitney U test was used for variable comparison. A two-sided *P* value<0.05 was considered significant.

## 3 Results

### 3.1 Feature extraction and model construction of Models 1-3

#### 3.1.1 General patient information

The difference in age between the 3 groups was statistically significant, and two-by-two ANOVA showed that the average age of the MLN group [(41.8 ± 12.2)] was significantly lower than that of the HLN group [(49.9 ± 11.7)] and non-MLN group [(46.9 ± 11.2)] (*P*=0.003, *P*<0.001). The difference in the age between the HLN and non-MLN groups was not statistically significant (*P*=0.064). There was a statistically significant difference in the sex distribution among all 3 groups (χ²=19.838, *P*<0.001), a statistically significant difference in the sex distribution between the MLN and HLN groups (χ²=15.635, *P*=0.015), and no statistically significant difference in the sex distribution between the HLN and non-MLN groups (χ²=8.634, *P*= 0.064). In this study, lymph nodes were included with a short diameter of 5.0 mm to 16.3 mm and a long diameter of 5.2 mm to 19.3 mm.

We used the stratified random sampling method to divide patients into training and test groups at a 7:3 ratio. In model 1, there were 115 patients in the training group (MLN group: 48 patients; HLN group: 67 patients) and 49 patients in the test group (MLN group: 23 patients; HLN group: 26 patients). In model 2, there were 125 patients in the training group (48 patients in the MLN group and 77 patients in the non-MLN group) and 53 patients in the test group (23 patients in the MLN group and 30 patients in the non-MLN group). In model 3, there were 140 patients in the training group (72 patients in the non-MLN group and 68 patients in the HLN group) and 60 patients in the test group (35 patients in the non-MLN group and 25 patients in the HLN group).

In the training group, there were statistically significant differences in age, CT value of the arterial phase and CT value difference between the venous phase and arterial phase in model 1. In model 2, there were statistically significant differences in age, sex distribution, short and long diameter of the lymph nodes, CT value of the arterial phase, CT value of the venous phase, CT value difference between venous phase and arterial phase and lymph node shape. In model 3, there were statistically significant differences in the long diameter of the lymph node, CT value of the venous phase, CT value difference between the venous phase and arterial phase and lymph node shape, as shown in **Table 1**.

**Table 1:** Comparison of clinical characteristics of the three models in the training group.

Variable	Model 1			Model 2			Model 3		
	MLN(n=48)	HLN(n=67)	*P* value	MLN(n=48)	non-MLN(n=77)	*P* value	non-MLN(n=72)	HLN(n=68)	*P* value
Age(mean±SD)	41.3±11.9	49.2±12.	<0.001	40.9±12.7	46.6±11.0	0.008	47.2±10.6	49.2±12.2	0.196
Sex(n, %)			0.065			0.016			0.693
Male	14 (29.2)	9(13.4)		13(27.1)	7(9.1)		6(8.3)	8(11.8)	
Female	34 (70.8)	58(86.6)		35(72.9)	70(90.9)		66(91.7)	60(88.2)	
Short diameter of lymph node(mm)	6.4 ± 2.3	5.8 ± 1.1	0.051	6.4 ± 2.3	5.8 ± 1.1	0.036	5.5 ± 0.6	5.7 ± 1.1	0.109
Long diameter of lymph node(mm)	9.0 ± 4.1	8.4 ± 2.1	0.366	9.0 ± 4.1	7.8 ± 2.1	0.029	7.4 ± 1.7	8.3 ± 2.1	0.004
CT value of arterial phase	118.2 ± 49.7	84.5 ± 16.3	<0.001	119.0 ± 52.7	81.0 ± 18.9	<0.001	81.3 ± 18.5	86.6 ± 16.9	0.079
CT value of venous phase	122.7 ± 36.8	114.7 ± 22.2	0.147	123.8 ± 37.6	101.8 ± 20.4	<0.001	102.9 ± 19.9	116.4 ± 21.9	<0.001
CT difference between venous phase and arterial phase	4.5 ± 30.5	30.2 ± 17.7	<0.001	4.9 ± 31.7	20.8 ± 14.7	<0.001	21.6 ± 14.1	29.8 ± 17.5	0.002
Shape			0.594			0.033			0.009
Regular	36(75.0)	46(68.7)		37(77.1)	71(92.2)		66(91.7)	50(73.5)	
Irregular	12(25.0)	21(31.3)		11(22.9)	6(7.8)		6(8.3)	18(26.5)	

#### 3.1.2 Radiomics feature extraction and selection and radiomics model establishment

**Figure 5:**
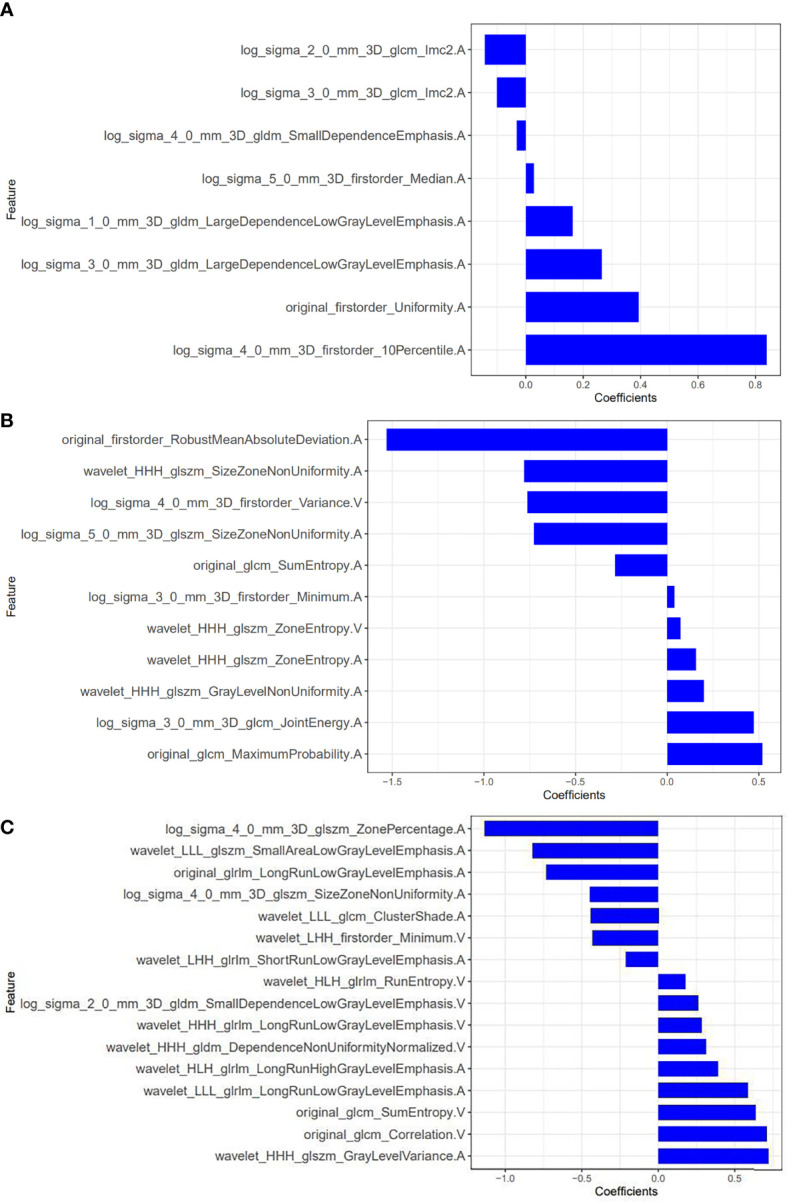
Radiomic features screened by models 1-3 and their weights. **(A)** model 1; **(B)** model 2; **(C)** model 3 (The letters A and V in the feature names indicate that the feature was extracted from the arterial phase and the venous phase, respectively).


Eight optimal features were selected from model 1, all of which were from the arterial phase. There were 3 first-order statistical features, 2 gray level cooccurrence matrix (GLCM) features and 3 gray level dependence matrix (GLDM) features. Eleven features were screened from model 2, among which 9 were from the arterial phase and 2 were from the venous phase. There were 3 first-order statistical features, 5 gray level size zone matrix (GLSZM) features and 3 GLCM features. Sixteen features were screened from model 3, of which 9 were from the arterial phase and 7 were from the venous phase (**Figure 5**). There was 1 first-order statistical feature, 3 GLCM features, 4 GLSZM features, 2 GLDM features and 6 gray level run length matrix (GLRLM) features. Based on these features and their corresponding regression coefficients, the radiomics model was constructed, and the Radscore was formulated as follows:

$$\text{Radscore}=\sum_{\text{n}}^{1}feature_{i}*{\text{coefficient}}_{\text{i}}+\text{Compensation coefficient}$$

Model 1: Radscore=0.839*log_sigma_4_0_mm_3D_firstorder_10Percentile. A+ -0.031*log_sigma_4_0_mm_3D_gldm_SmallDependenceEmphasis.A+0.393*original_firstorder_Uniformity.A+-0.1*log_ sigma_3_0_mm_ 3D_glcm_Imc2. A+ 0.163*log_sigma_1_0_mm_3D_gldm_LargeDependenceLowGrayLevelEmphasis.A+0.028*log_sigma_5_0_mm_3D_firstorder_Median.A+0.265*log_sigma_3_0_mm_3D_gldm_LargeDependenceLowGrayLevelEmphasis.A+-0.142*log_sigma_2_0_ mm_ 3D_glcm_ Imc2.A+0.443

Model 2: Radscore=-0.78*wavelet_HHH_glszm_SizeZoneNonUniformity. A+ 0.472*log_sigma_3_0_mm_3D_firstorder_Minimum.A+0.158*wavelet_HHH_glszm_ZoneEntropy.A+-1.531*original_firstorder_RobustMeanAbsoluteDeviation. A+ 0.201*wavelet_HHH_glszm_GrayLevelNonUniformity.A+0.039*log_sigma_3_0_mm_3D_firstorder_Minimum.A+-0.763*log_ sigma_4_0_mm_3D_ firstorder_ Variance. V+0.518*original_glcm_MaximumProbability.A+-0.727*log_ sigma_5_0_ mm_3D_glszm_SizeZoneNonUniformity.A+0.072*wavelet_HHH_glszm_ZoneEntropy.V+-0.284*original_glcm_SumEntropy.A+1.013

Model 3: Radscore=-0.822*wavelet_LLL_glszm_SmallAreaLowGrayLevel -Emphasis.A+0.718*wavelet_HHH_glszm_GrayLevelVariance.A+0.443*wavelet_LLL_glcm_ClusterShade.A+-0.73*original_glrlm_ LongRunLowGrayLevelEmphasis. A+-1.132* log_sigma_ 4_0_mm_3D_glszm_ ZonePercentage. A+ 0.388*wavelet_ HLH_glrlm_LongRunHighGrayLevelEmphasis.A+0.31*wavelet_HHH_gldm_DependenceNonUniformityNormalized.V+-0.209* wavelet_LHH_glrlm_ ShortRunLow GrayLevelEmphasis.A+0.176*wavelet_HLH_glrlm_RunEntropy.V+0.705*original_glcm_Correlation.V+-0.444*log_sigma _4_0_mm_3D_glszm_ SizeZoneNon Uniformity.A+0.585*wavelet_LLL_glrlm_LongRunLowGrayLevelEmphasis.A+-0.43*wavelet_LHH_firstorder_Minimum.V+0.28*wavelet_HHH_glrlm_LongRunLowGrayLevelEmphasis.V+0.634*original_glcm_SumEntropy.V+-0.465

The diagnostic efficacy of radiomics models 1-3 is shown in **Table 2**.

**Table 2:** Diagnostic efficacy of the radiomics models of models 1-4.

Evaluation indicators	Model 1		Model 2		Model 3		Model 4	
	Training group	Test group	Training group	Test group	Training group	Test group	Training group	Test group
AUC	0.97	97.92	0.97	0.85	0.93	0.74	0.94	0.93
Accuracy (%)	0.93	91.30	88.00	67.92	88.57	71.67	89.47	87.65
Sensitivity (%)	93.91	98.39	80.52	70.00	92.65	76.00	89.78	90.48
Specificity (%)	89.79	92.00	93.75	86.96	84.72	68.57	88.68	77.78
Positive predictive value (%)	91.04	88.68	100.00	84.21	85.14	63.33	95.35	93.44
Negative predictive value (%)	88.46	87.50	76.19	58.82	92.42	80.00	77.05	70.00

#### 3.1.3 Clinical feature screening and nomogram construction

**Figure 6:**
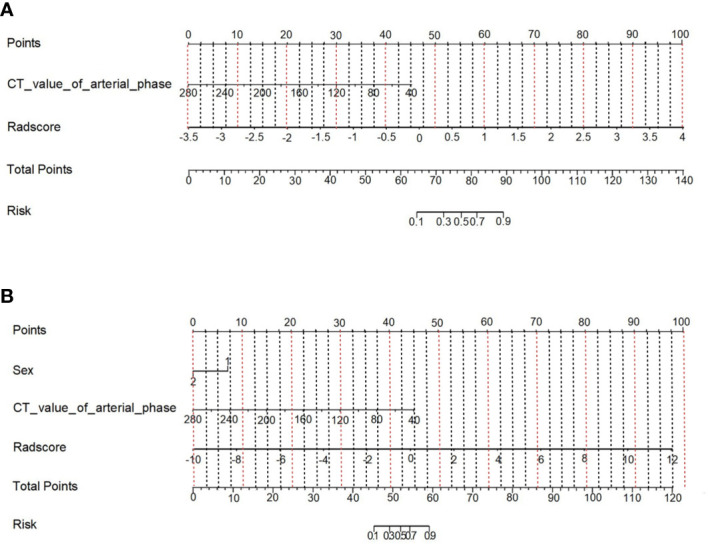
Nomogram of the prediction model combining clinical risk factors and CT radiomics features. **(A)** Model 1; **(B)** Model 2.

Multivariate logistic regression analysis showed that the CT value in the arterial phase (OR=1.05, 95% CI: 1.02~1.09) was an independent clinical risk factor for model 1. Sex (OR=0.1, 95% CI: 0.01-0.67) and CT value in the arterial phase (OR=0.96, 95% CI 0.94-0.98) were independent clinical risk factors for model 2. Combining the clinical risk factors and radiomics labels, a nomogram was established, and the corresponding scores of each predictive index were obtained, which were then summed and finally reflected by the total score. The constructed nomogram is shown in **Figure 6**. There were no statistically significant clinically relevant risk factors in model 3, so no nomogram was constructed.

### 3.2 Feature extraction and model construction of model 4

**Figure 7:**
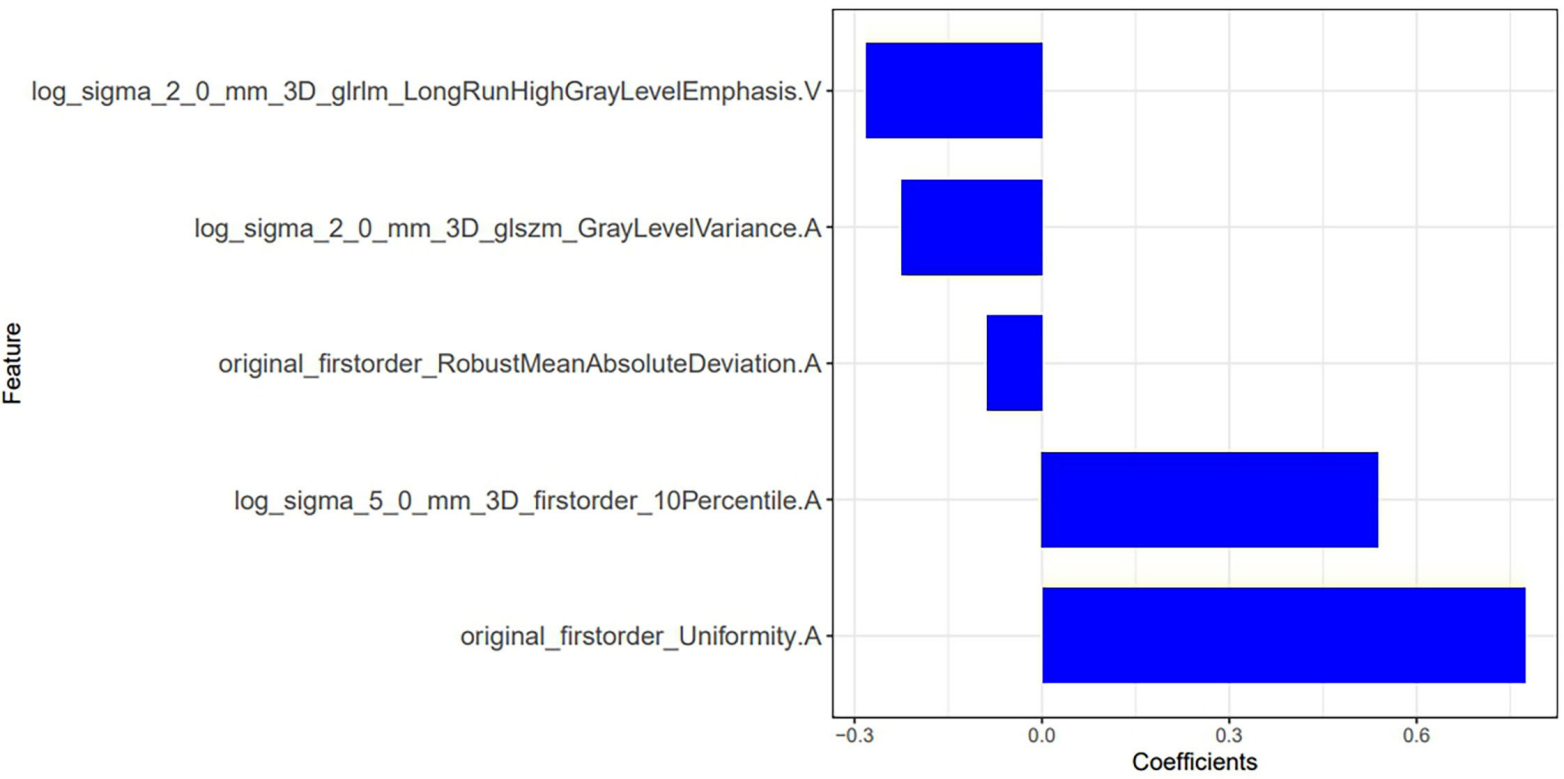
Radiomic features screen by model 4 and their weights.

**Figure 8:**
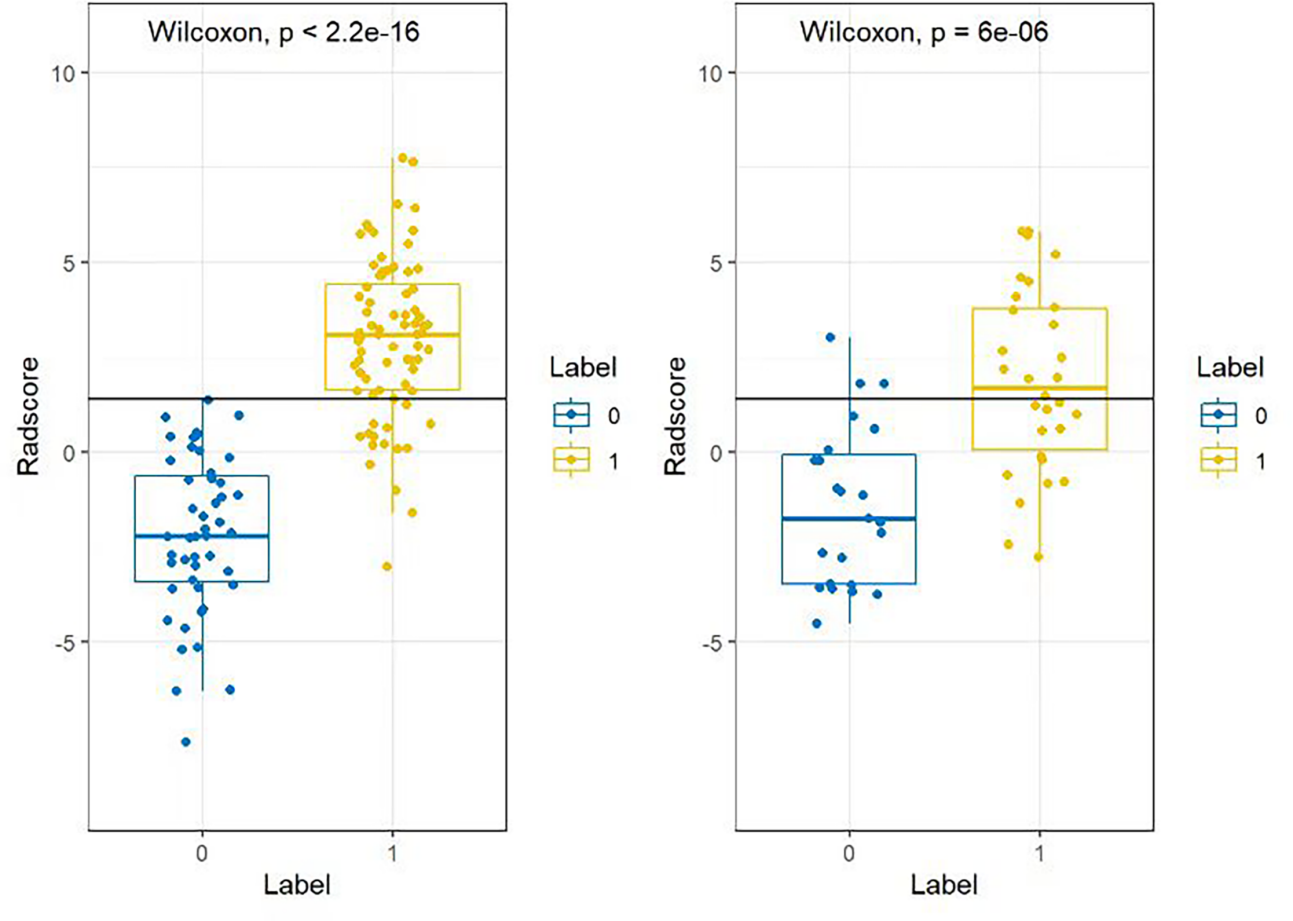
Box plot of the radiomics scores of the training group and the test group in model 4. Blue (Label 0) represents the MLN group, and yellow (Label 1) represents the HLN and non-MLN group. The difference between the two groups was statistically significant (*P<* 2.2^e-16^).

In the training group, after feature extraction with mRMR and LASSO, 5 radiomics features with strong correlations were ultimately identified (**Figure 7**). Four features were from the arterial phase, and one was from the venous phase. Based on these features and their corresponding regression coefficients, a radiomics model was constructed. There were 3 first-order statistical features, 1 GLSZM feature and 1 GLRLM feature. The box diagram shows that in the training group and the test group, the difference in the radiomics score between patients in the MLN group and in the non-MLN + HLN) was statistically significant (**Figure 8**).

Model 4: Radscore=0.773*original_firstorder_Uniformity.A+0.539*log_sigma_ 5_0_mm_3D_firstorder_10Percentile.A+-0.226*log_ sigma_2_0_mm_3D_ glszm_ GrayLevelVariance.A+-0.089*original_ firstorder_ RobustMeanAbsolute Deviation. A+-0.282*log_ sigma_2_0_mm_ 3D_glrlm_Long RunHighGrayLevel Emphasis. V+ 1.279

The diagnostic efficacy of radiomics model 4 is shown in **Table 2**.

**Figure 9:**
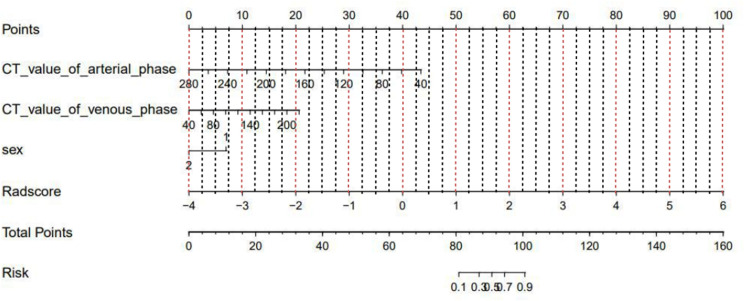
Nomogram of model 4 constructed by combining clinical risk factors and CT radiomics features.

Multivariate logistic regression analysis revealed that sex (OR=0.21, 95% CI: 0.05-0.93), CT value in the arterial phase (OR=0.96, 95% CI: 0.93-0.99) and CT value in the venous phase (OR=1.03, 95% CI of 1.00-1.05) were independent clinical risk factors for discriminating between the MLN and (HLN+ non-MLN) groups. A nomogram was established combining the clinical risk factors and radiomic features (**Figure 9**).

### 3.3 ROC curve analysis, calibration curve analysis and DCA for evaluating the efficacy and value of the nomograms

**Figure 10:**
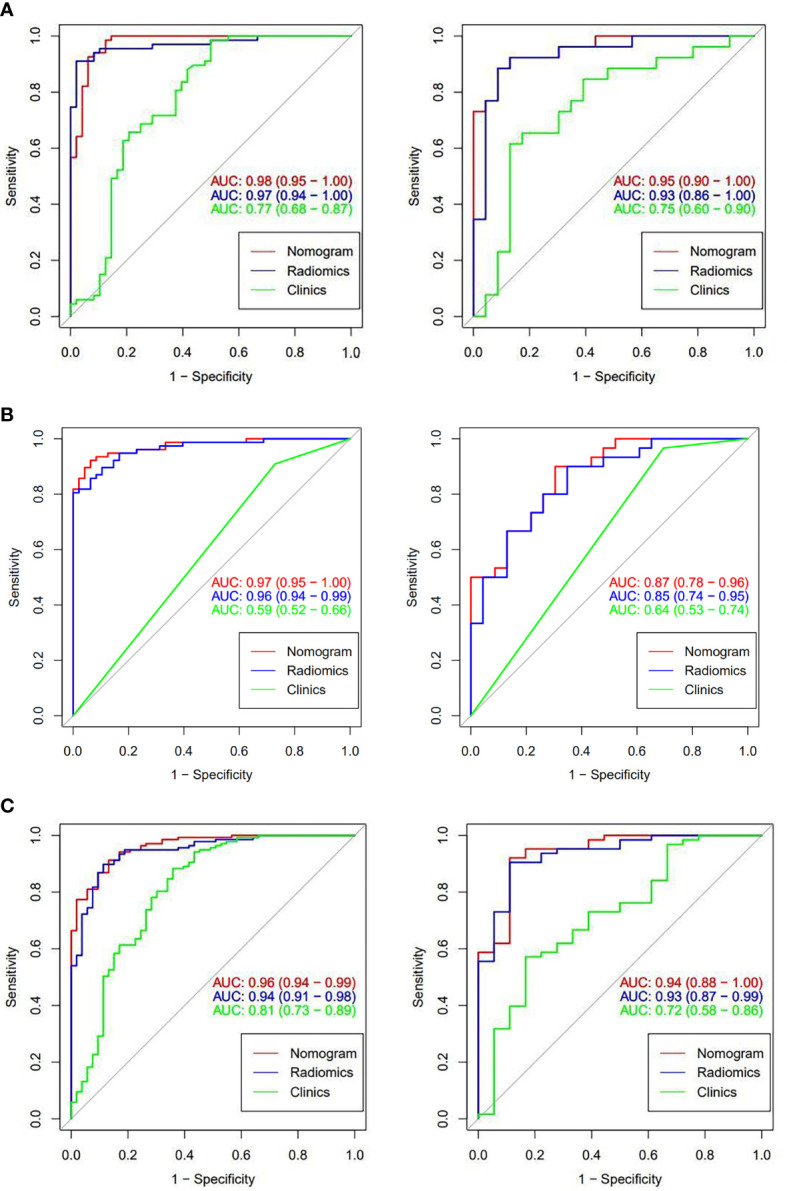
ROC curves of models 1, 2, and 4 for the corresponding classification efficacy of the radiomics models, clinical features, and nomogram models in the training group and test group. (**A** are the training group and test group of model 1, respectively; **B** are the training group and test group of model 2; and **C** are the training group and test group of model 4).

ROC curve analysis was used to assess the diagnostic efficacy of the three models (**Figure 10**). In the training group, the AUC values of the nomogram model were higher than those of the radiomics model and clinical model. DeLong’s test showed that there were no significant differences in the AUC values between the nomogram model and the radiomics model for models 1, 2, and 4 (model 1: Z=2.1482, *P*=0.062; model 2: Z=1.637, *P*=0.102; model 4: Z=7.463, *P*=0.132). The differences between the nomogram model and clinical model were statistically significant (model 1: Z=4.491, *P*< 0.001; model 2: Z=10.376, *P*<0.001; model 4: Z=3.140, *P*=0.002). In the test group, the diagnostic efficacy of the nomogram model was higher than that of the radiomics model and clinical model. DeLong’s test showed that there were no significant differences between the nomogram and radiomics model for model 1, model 2, and model 4 (model 1: Z=2.945, *P*=0.179; model 2: Z=3.599, *P*=0.086; model 4: Z= 8.051, *P*=0.142), and the differences between the nomogram and clinical model were statistically significant (model 1: Z=1.448, *P*=0.033; model 2: Z=1.345, *P*=0.019; model 4: Z=3.943, *P*< 0.001). The diagnostic efficacy of the radiomics model, clinical model and nomogram model for models 1, 2 and 4 are shown in **Table 3**.

**Table 3:** Diagnostic efficacy of radiomics model, clinical model, nomogram model of model 1, 2, 4.

model 1	Training group				Test group			
	AUC	Accuracy (%)	Sensitivity (%)	Specificity (%)	AUC	Accuracy (%)	Sensitivity (%)	Specificity (%)
radiomics model	0.97	94.91	91.04	97.92	0.93	89.8	88.46	91.3
clinical model	0.77	78.26	98.51	50	0.75	63.27	76.58	30.43
nomogram model	0.98	93.04	92.54	93.75	0.95	89.8	88.89	90.91
model 2								
radiomics model	0.97	88.00	80.52	93.75	0.85	67.92	70	86.96
clinical model	0.59	66.4	90.91	27.08	0.64	67.92	96.67	30.43
nomogram model	0.97	92	88.31	97.92	0.87	75.47	84	67.86
model 4								
radiomics model	0.94	89.47	89.78	88.68	0.93	87.65	90.48	77.78
clinical model	0.81	81.58	88.32	64.15	0.72	72.84	84.13	33.33
nomogram model	0.96	90	91.24	86.79	0.94	92.59	95.24	83.33

**Figure 11:**
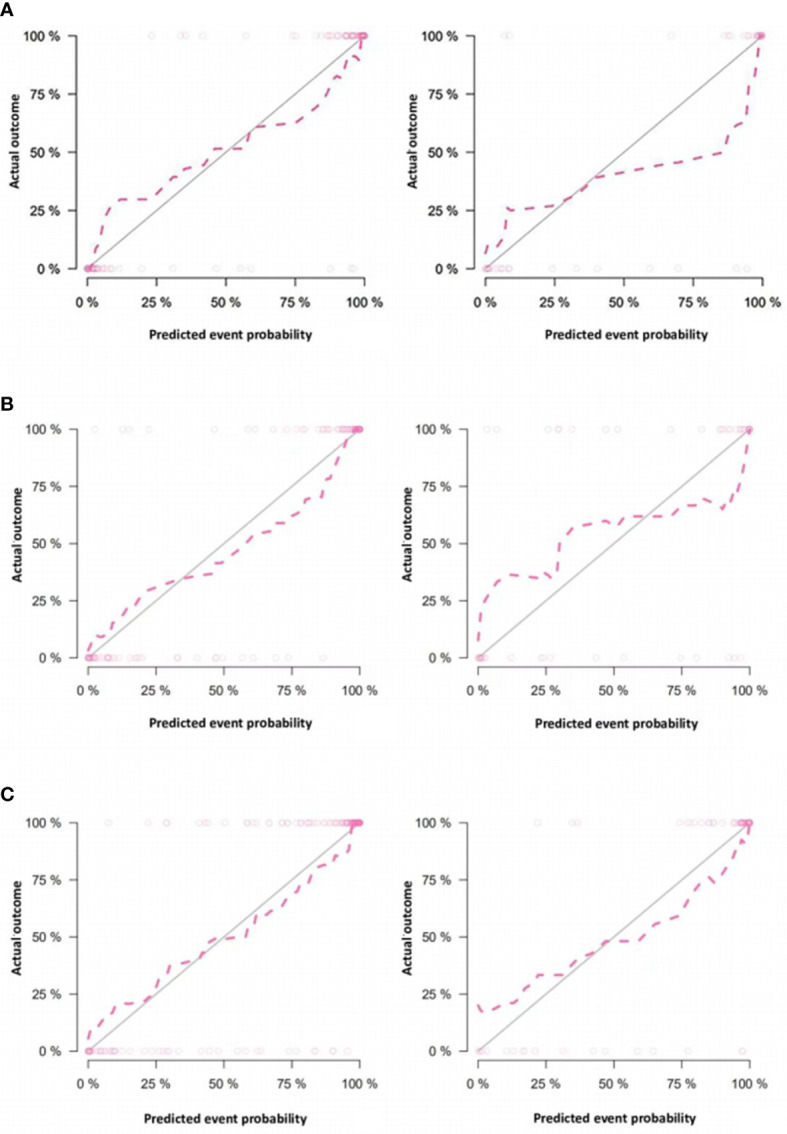
Calibration curves of the nomograms in the training group and test group of models 1, 2 and 4. (**A** are the training group and test group of model 1, respectively; **B** are the training group and test group of model 2; and **C** are the training group and test group of model 4) In the calibration curve, the horizontal axis represents the predicted model value, and the vertical axis represents the real value. The prediction efficacy is better if the red line is closer to the gray line.

**Figure 12:**
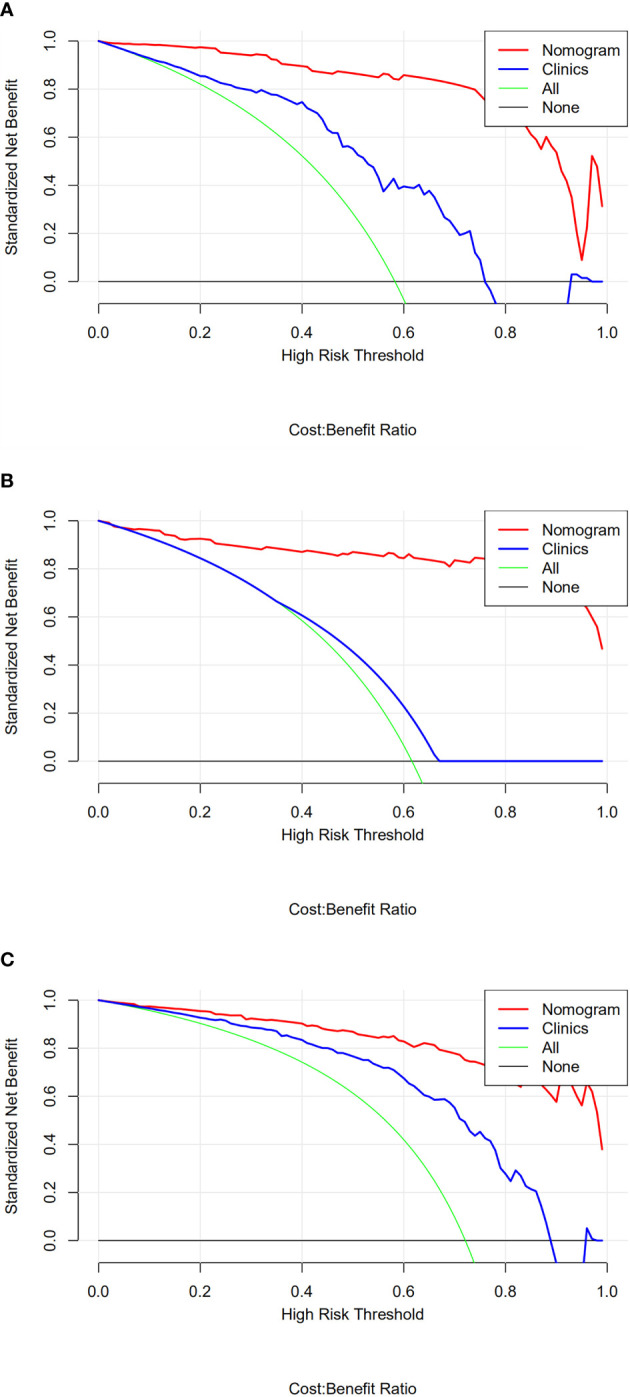
**(A-C)** are the decision curves of nomogram models 1, 2, and 4 for the corresponding lymph node classification in the test group, respectively. The red line in the figure represents the diagnostic nomogram model built from the imaging features, and the blue line represents the diagnostic nomogram model built from the clinical features. The green line represents the hypothesis that all patients had lymph node metastasis; the black lines running across the bottom are assuming that none of the patients had lymph node metastasis. The potential clinical benefits of the radiomics-based models are consistently higher than those of the other three models.

The calibration curves of nomogram prediction models 1, 2, and 4 in the training group and test group showed good predictive efficacy (**Figure 11**). The calibration curve of model 4 is closer and better fits to the corresponding diagonal line than that of models 1 and 2. DCA showed that the nomograms of the 3 models outperformed the clinical model across all assessed risk thresholds (**Figure 12**).

## 4 Discussion

Currently, noninvasive assessment of the nature of lymph nodes mainly relies on imaging features. Ultrasound, as the main imaging method for examining the thyroid, has important value in the diagnosis of thyroid diseases and cervical lymph nodes (13, 14). However, its diagnostic accuracy is affected by the sonographer’s subjectivity and diagnostic experience. CT has advantages in the evaluation of central and superior mediastinal lymph nodes, but it is limited by the need for morphological changes in the lymph nodes to diagnose metastasis. It has been reported that both ultrasound and CT have a sensitivity below 50% in diagnosing central lymph node metastasis (15). The diffusion-weighted MR imaging sequence has certain value in judging the condition of the lymph nodes, but for smaller lymph nodes, the misdiagnosis and missed diagnosis rates are high, especially when the short diameter is less than 10 mm. In recent years, many studies have demonstrated the increasing value of radiomics in determining the condition of lymph nodes. Onoue et al. (16) showed that radiomics based on CT can distinguish metastatic lymph nodes from PTC, tuberculosis, and oropharyngeal squamous cell carcinoma with significantly higher diagnostic accuracy than two neuroradiologists. Seidler et al. (17) found that machine learning texture analysis based on dual-energy CT helped to distinguish different pathological lymph nodes (metastatic head and neck squamous cell carcinoma lymph nodes, lymphoma, inflammation) and normal lymph nodes with higher accuracy. This study investigated the stratified predictive value of a radiomics model based on CT images for metastatic lymph nodes, nonmetastatic lymph nodes, and reactive hyperplastic lymph nodes of benign lesions among patients with PTC to provide guidance for treatment.

In this study, patient age, sex, and lymph node CT signs (long diameter, short diameter, arterial phase CT value, venous phase CT value, arterial and venous phase CT difference value, and lymph node morphology) were statistically different across multiple models through one-way ANOVA, indicating that clinical features and conventional CT images are of value in the identification of lymph nodes with PTC metastasis. However, the morphological signs of early metastatic lymph nodes are often atypical, and the sensitivity in diagnosis is relatively low. The interpretation of image features depends on the clinical experience of the radiologist and is subjective, and thus there is a need to incorporate objective, quantitative indicators to assist in diagnosis. Therefore, the nomogram combines clinical features with objective radiomics features to improve diagnostic efficacy.

The best feature sets identified by the four models in this study all included first-order statistical features and texture features, the latter of which accounted for the higher proportion. The first-order features can quantitatively reflect the global voxel intensity distribution of the ROI and then evaluate the overall information of the lymph nodes. Texture features can describe the spatial distribution of pixel intensity in images and reflect the histological types and pathological properties of lesions with high sensitivity (18). The combination of the two can help comprehensively evaluate the heterogeneity of lymph nodes from different perspectives. Compared with nonmalignant lymph nodes, malignant lymph nodes have more abnormal new blood vessels, increased cell permeability and internal necrosis, which will change the roughness of lymph nodes, resulting in heterogeneity. The above changes are not easily detected by the naked eye but can be reflected by texture features, which are not affected by subjective factors (19).

In this study, 8, 11, 16 and 5 of the best radiomics features were selected for the 4 models by LASSO regression analysis. Among them, the all features of model 1 (PTC metastatic lymph nodes and nonmetastatic lymph nodes), nine of the features of model 2 (PTC metastatic lymph nodes and nonmetastatic lymph nodes), and four of the features of model 4 (PTC metastatic lymph nodes and nonmalignant lymph nodes) were from the arterial phase, suggesting that compared with those of the venous phase, the radiomics features of the arterial phase have higher diagnostic value in distinguishing malignant lymph nodes from nonmalignant lymph nodes of PTC. Xu et al. (20) found that radiomics features extracted from dual-energy CT arterial phase-weighted fusion images can effectively diagnose PTC cervical lymph node metastasis, and the three radiomics features screened were all from the arterial phase. Zhao et al. (21) found that the model based on texture features extracted from arterial-phase CT images was more advantageous in evaluating the lymph node metastasis in PTC, with a higher diagnostic coincidence rate (75.47%) than the model built from the features extracted from the venous phase (71.69%). Consistent with the results of this study, the reason may be that metastatic lymph nodes have a more abundant blood supply and more obvious early enhancement among patients with PTC (22). Unlike other models, 9 of the 16 features in model 3 (PTC nonmetastatic lymph nodes and reactive hyperplastic lymph nodes in benign lesions) were from the arterial phase, and 7 were from the venous phase. The reason may be that the blood supply of nonmalignant lymph nodes in the venous phase is enhanced, while the enhancement in the arterial phase is relatively weaken. Therefore, the number of venous phase features extracted for the lymph nodes in model 3 was substantially increased. Park et al. (12) confirmed that arterial phase CT scans can improve the diagnostic accuracy for PTC lymph node metastasis compared with venous phase CT, which is often used to evaluate lymph nodes of other pathological types, such as squamous cell carcinoma and tuberculosis lymph nodes.

A larger short lymph node diameter and central necrosis are usually considered indications for malignant transformation, while the short diameter is proportional to the rate of metastasis. The differences between metastatic lymph nodes and nonmalignant lymph nodes and the differences between nonmetastatic lymph nodes and benign reactive lymph nodes in the models in this study were not related to the short lymph node diameter, which is consistent with the research results of Li et al. (23). Ren et al. (24) found that the difference in the short diameter between positive and negative lymph nodes in early tongue cancer pathological metastasis was statistically significant, suggesting that the short diameter of the lymph nodes has a certain reference value for the diagnosis of occult metastasis. In this study, the short lymph node diameter was statistically significant in one-way ANOVA but not in multivariate logistic regression analysis. This suggests that the short diameter of the lymph nodes may be valuable in distinguishing metastatic and nonmetastatic lymph nodes, but not to a significant degree; the reason may be that the nonmalignant lymph node group in our study included nonmetastatic lymph nodes and reactive hyperplastic lymph nodes. Reactive hyperplastic lymph nodes may be significantly enlarged, which reduces the difference in lymph node diameter between the nonmalignant and metastatic groups.

The nomogram in this study was established based on radiomics features and clinical data. Nomograms can more intuitively and individually evaluate the nature of lymph nodes than their corresponding models. Verification of the effectiveness of the models in this study revealed a number of findings. In the first part, the nomograms of models 1 and 2 show high diagnostic performance in both the training and test groups, higher than the performance of the models built from radiomics labels or CT imaging features alone. Our study showed that the nomograms had high predictive efficacy and encompassed the advantages of integrating CT image features and radiomics. In this study, the arterial phase CT enhancement values of models 1 and 2 were independent clinical risk factors for judging the nature of lymph nodes, suggesting that there are certain differences in early enhancement between metastatic lymph nodes, nonmetastatic lymph nodes, and reactive hyperplastic lymph nodes. The degree of enhancement in the arterial phase has a certain value in differentiating the groups. Radiomics model 3 also showed high diagnostic performance in the training group and test group. After univariate and multivariate analyses, there were no clinically relevant risk factors between the two groups of lymph nodes in model 3, suggesting that there may be some heterogeneity in the internal radiomics characteristics of the two groups; however, this heterogeneity is low and cannot be detected with clinical and routine imaging examinations. The second part of this study summarized PTC nonmetastatic lymph nodes and benign reactive hyperplastic lymph nodes and built a predictive model for differentiating the two groups. The predictive model showed high discriminative ability, similar to the diagnostic value between separate groups. This indicates that there is no significant difference between the nonmetastatic lymph nodes of PTC and reactive hyperplastic lymph nodes, and simple binary classification can also achieve high diagnostic performance. In model 4, the CT value in the arterial phase was also an independent clinical risk factor, consistent with the results of models 1 and 2, suggesting that early lymph node enhancement plays an important role in distinguishing benign and malignant lymph nodes. As in models 1 and 2, the CT value in the venous phase was also an independent risk factor in model 4. The reason may be that the proportion of blood supply in the venous phase of nonmalignant lymph nodes is higher than that in the arterial phase. In this model, the proportion of nonmalignant lymph nodes increased, so the weight of the venous phase in the identification of the two groups increased.

ROC curve and calibration curve analyses reflect the diagnostic value of the model, and DCA reflects its clinical value (25). In this study, DCA was used to evaluate the clinical effectiveness of the model, which increased its credibility. Models 1, 2 and 4 provided a clear net benefit over the entire risk threshold range, suggesting that the models have certain clinical value.

The limitations of this study are as follows. (1) In this study, the ROIs were manually outlined by doctors with high accuracy, but due to subjectivity, the repeatability of these segmentations could be poor. Although we used a high ICC as a criterion for improving the consistency of the features, this low repeatability inevitably impacted the results. To make the model more robust and more suitable for clinical application, we will attempt to solve this problem by using semiautomatic or fully automatic segmentation using consensus contours in subsequent studies. (2) The sample size was small and drawn from a single center. The sample size will be expanded, and multicenter research and external validation research will be carried out in the future to improve the efficacy of the model. (3) In this study, only solitary thyroid nodules with a pathological result of PTC or adenoma were included. The pathological types were relatively singular; subsequent studies on the lymph nodes of lesions with different pathological types are needed to expand the scope of adaptation. Since we could not accurately judge the status of each lymph node before surgery, we adopted an all-or-nothing approach to select target lymph nodes according to postoperative pathological results. This method of lymph node selection is accurate, but due to the strict inclusion criteria, the sample size of the included lymph nodes is reduced.

In conclusion, the CT-enhanced nomogram performed well in predicting metastatic lymph nodes in the central cervical region and nonmalignant lymph nodes in patients with thyroid nodules and can provide guidance for clinical decision-making. The radiomics model showed high diagnostic efficacy in distinguishing nonmetastatic lymph nodes from benign lymph nodes, but there was no significant difference in the clinical features between the groups. There may be some heterogeneity between the two groups of lesions, but its degree was insufficient to produce significant differences on the basis of radiomics. Further experimental studies are needed.

## Data availability statement

The original contributions presented in the study are included in the article/supplementary material. Further inquiries can be directed to the corresponding author.

## Ethics statement

Ethical review and approval was not required for the study on human participants in accordance with the local legislation and institutional requirements. The patients/participants provided their written informed consent to participate in this study.

## Author contributions

Author 1(first author): Case collection, data collection and analysis, writing – first draft; Author 2: Data collection, writing - first draft; Author 3: Delineation of the region of interest; Author 4: Data analysis, writing, editing Author 5: Software, validation Author 6:(Corresponding author): Design, supervision, writing-review and editing of experimental methods. All authors contributed to the article and approved the submitted version
